# Hydrophobia from the Bite of a Pole-Cat

**Published:** 1859-02

**Authors:** 


*Hydrophobia from the Bite of a Pole-Cat.*—The (Augusta) Southern Medical and Surgical Journal, for January, contains the report of a case of hydrophobia, from the bite of a pole-cat, by Dr. R. De Jernett, of Greenville, Texas. The patient was a little girl, ten years of age, who was bitten on the 8th of January [1857, we presume,] and the symptoms of hydrophobia appeared Feb. 21st. She died on the 24th, with the charrcteristie symptoms. Tracheotomy was performed, but without relief.

